# Supplementary material for: Phytophthora Diversity in Pennsylvania Nurseries and Greenhouses Inferred from Clinical Samples Collected over Four Decades
Source: Microorganisms. 2020 Jul 16;8(7):1056. doi: 10.3390/microorganisms8071056 (PMC7409235; doi:10.3390/microorganisms8071056)
Supplement: Supplementary file 1 [file microorganisms-08-01056-s001.zip › Supplementary legends.doc]

Supplementary Table S1: Low count genera sampled in this study.

*The host for these isolates was not available.

Supplementary Table S2: Plants associated with Clade 1 *Phytophthora* species.

^1^ Potential new hosts are marked with an *.

Supplementary Table S3: Plants associated with Clade 2 species.

^1^ Potential new hosts are marked with an *.

Supplementary Table 4: Plants associated with Clades 4 and 5 species.

^1^ Potential new hosts are marked with an *.

Supplementary Table S5: Plants associated with Clade 6 species.

^1^ Potential new hosts are marked with an *.

Supplementary Table S6: Plants associated with Clade 7 species.

^1^ Potential new hosts are marked with an *.

Supplementary Table S7: Plants associated with Clade 8 species.

^1^ Potential new hosts are marked with an *.

Supplementary Table S8: Plants associated with Clade 9 species.

^1^ Potential new hosts are marked with an *.

Supplementary Table S9: *Phytophthora* species found by county. Numbers in parenthesis indicate the number of isolates found for each species.

^1^The origins of the samples in this group are not available.

Supplementary Figure S1: Clade 1 isolates recovered per year. Because relatively few isolates were found in 1975-1984, these are grouped into a single category.

Supplementary Figure S2: Tissue of origin for Clade 1 species. The “stem/leaves” category denotes isolates that were recovered from tissue that may correspond to either category.

Supplementary Figure S3: ML phylogenetic tree of representative Clade 1 isolates with reference sequences of same and closely related species. Bootstrap proportions are displayed at branch nodes. Branch length is proportional to genetic distance with the scale displayed at the bottom on the tree. *P. nemorosa* is used as an outgroup.

Supplementary Figure S4: Clades 2, 2A, and 2B isolates recovered per year.

Supplementary Figure S5: Tissue of origin for Subclades 2, 2A and 2B species.

Supplementary Figure S6: ML phylogenetic tree of Clade 2 isolates with reference sequences of same and closely related species. Bootstrap proportions are displayed at branch nodes. Branch length is proportional to genetic distance with the scale displayed at the bottom on the tree. *P. nemorosa* is used as an outgroup.

Supplementary Figure S7: Subclade 2C isolates recovered per year.

Supplementary Figure S8: Tissue of origin for Subclade 2C species.

Supplementary Figure S9: Clade 4 isolates recovered per year.

Supplementary Figure S10: Tissue of origin for Clade 4 species.

Supplementary Figure S11: ML phylogenetic tree of Clade 4 isolates with reference sequences of same and closely related species. Bootstrap proportions are displayed at branch nodes. Branch length is proportional to genetic distance with the scale displayed at the bottom on the tree. *P. nemorosa* is used as an outgroup.

Supplementary Figure S12: ML phylogenetic tree of Clade 5 isolates with reference sequences of same and closely related species. Bootstrap proportions are displayed at branch nodes. Branch length is proportional to genetic distance with the scale displayed at the bottom on the tree. *P. nemorosa* is used as an outgroup.

Supplementary Figure S13: Clade 6 isolates recovered per year.

Supplementary Figure S14: Tissue of origin for Clade 6 species.

Supplementary Figure S15: ML phylogenetic tree of Clade 6 isolates with reference sequences of same and closely related species. Bootstrap proportions are displayed at branch nodes. Branch length is proportional to genetic distance with the scale displayed at the bottom on the tree. *P. nemorosa* is used as an outgroup.

Supplementary Figure S16: Subclades 7A and 7B isolates recovered per year.

Supplementary Figure S17: Tissue of origin for Subclades 7A and 7B species.

Supplementary Figure S18: Clade 7 *P. cinnamomi* subgroup isolates recovered per year.

Supplementary Figure S19: Tissue of origin for Clade 7 *P. cinnamomi* subgroup species.

Supplementary Figure S20: ML phylogenetic tree of Clade 7 isolates with reference sequences of same and closely related species. Bootstrap proportions are displayed at branch nodes. Branch length is proportional to genetic distance with the scale displayed at the bottom on the tree. *P. nemorosa* is used as an outgroup.

Supplementary Figure S21: Clade 8A isolates recovered per year.

Supplementary Figure S22: Tissue of origin for Clade 8A species.

Supplementary Figure S23: ML phylogenetic tree of Clade 8 isolates with reference sequences of same and closely related species. Bootstrap proportions are displayed at branch nodes. Branch length is proportional to genetic distance with the scale displayed at the bottom on the tree. *P. nemorosa* is used as an outgroup.

Supplementary Figure S24: Clade 9 isolates recovered per year.

Supplementary Figure S25: Tissue of origin for Clade 9 species.

Supplementary Figure S26: ML phylogenetic tree of Clade 9 isolates with reference sequences of same and closely related species. Bootstrap proportions are displayed at branch nodes. Branch length is proportional to genetic distance with the scale displayed at the bottom on the tree. *P. nemorosa* is used as an outgroup.

Supplementary Figure S27: A ML phylogenetic tree of PDA 312 with all known species used in this study. PDA 312 used gene markers for β-tub, HSP90, ITS and COX-2. *Halophytophthora fluvialis* is used as the outgroup. Bootstrap proportions are displayed at branch nodes. Branch length is proportional to genetic distance with the scale displayed at the bottom on the tree.

Supplementary Figure S28: Number of isolates and species by county.
