# Supplementary material for: Phytophthora Diversity in Pennsylvania Nurseries and Greenhouses Inferred from Clinical Samples Collected over Four Decades
Source: Microorganisms. 2020 Jul 16;8(7):1056. doi: 10.3390/microorganisms8071056 (PMC7409235; doi:10.3390/microorganisms8071056)
Supplement: Supplementary file 1 [file microorganisms-08-01056-s001.zip › Supplementary Table S1.doc]

Supplementary Table S1: Low count genera sampled in this study.

| Genera | Number of isolates recovered from each genus |
| --- | --- |
| *Buxus, Chamaedorea, Fragaria, Hydrangea, Iberis, Juniperus, Prunus, Thuja* | 3 |
| *Acer, Begonia x hiemalis, Citrullus, Cotoneaster, Dracaena, Helichrysum, Hibiscus, Juglans, Lamium, Lonicera, Mandevilla, Pelargonium, Rosmarinus, Sedum, Sorbus, Viburnum, N/A** | 2 |
| *"Conifer", Ajuga, Amelanchier, Brachteanthus , Bracteantha, Brassica, Castanea, Cedrus, Chionanthus, Cucumis, Daphne, Echinops , Enkianthus, Forsythia, Gardenia, Gypsophila, Heuchera, Huernia, Lactuca, Leucanthemum, Mangifera, Miscanthus, Myrica, Osteospermum, Oxydendrum, Pachysandra, Peperomia, Pescatoria, Pilea, Psidium, Quercus, Robinia, Rubus, Rudbeckia, Sassafras, Schlumbergera, Sinningia, Spinacia, Xanthocerus* | 1 |

*The host for these isolates was not available.
