# Supplementary material for: Phytophthora Diversity in Pennsylvania Nurseries and Greenhouses Inferred from Clinical Samples Collected over Four Decades
Source: Microorganisms. 2020 Jul 16;8(7):1056. doi: 10.3390/microorganisms8071056 (PMC7409235; doi:10.3390/microorganisms8071056)
Supplement: Supplementary file 1 [file microorganisms-08-01056-s001.zip › Supplementary Table S2.doc]

Supplementary Table S2: Plants associated with Clade 1 *Phytophthora* species.

| Species | Host^1^ | # of isolates |
| --- | --- | --- |
| *P. cactorum* (N=105) | *Abies* spp. | 37 |
|  | *Cornus florida* | 2 |
|  | *Cucurbita pepo* | 4 |
|  | *Daphne* sp*.* | 1 |
|  | *Fragaria anannassas* | 1 |
|  | *Fuchsia hybrida* | 1 |
|  | *Gypsophila paniculate* | 1 |
|  | *Hedera* sp*.* * | 1 |
|  | *Hydrangea quercifolia* * | 1 |
|  | *Juglans nigra* | 1 |
|  | *Kalmia latifolia* * | 1 |
|  | *Solanum lycopersicon* | 1 |
|  | *Malus* spp*.* | 28 |
|  | *Pinus* spp*.* | 2 |
|  | *Prunus persica* | 1 |
|  | *Pseudotsuga menziesii* | 2 |
|  | *Rhododendron* spp*.* | 17 |
|  | *Viola tricolor hortensis* | 2 |
|  | *Xanthocerus sorbifolia* * | 1 |
| *P. pseudotsugae* (N=1) | *Picea pungens* * | 1 |
| *P. hedraiandra* (N=2) | *Rhododendron* spp*.* | 2 |
| *P. nicotianae* (N=111) | *Antirrhinum* sp*.* | 2 |
|  | *Bacopa* sp*.* | 6 |
|  | *Buxus* sp*.* | 2 |
|  | *Cedrus libani* | 1 |
|  | *Chionanthus virginicus* | 1 |
|  | *Chrysanthemum* sp*.* | 1 |
|  | *Cornus florida* | 1 |
|  | *Dracaena* sp*.* | 1 |
|  | *Enkianthus campanulatus* | 1 |
|  | *Euphorbia* spp*.* | 4 |
|  | *Fragaria anannassas* | 2 |
|  | *Fuchsia hybrida* | 15 |
|  | *Gardenia* sp*.* | 1 |
|  | *Hibiscus* sp*.* | 2 |
|  | *Huernia zebrina* | 1 |
|  | *Lavandula* sp*.* | 12 |
|  | *Lonicera* sp*.* | 1 |
|  | *Solanum lycopersicon* | 10 |
|  | *Mandevilla* sp*.* | 2 |
|  | *Pachysandra procumbens* | 1 |
|  | *Peperomia* | 1 |
|  | *Petunia* sp*.* | 5 |
|  | *Picea pungens* | 1 |
|  | *Rhododendron spp.* | 11 |
|  | *Rosmarinus officinalis* | 1 |
|  | *Saintpaulia ionantha* | 9 |
|  | *Schlumbergera bridgesii* | 1 |
|  | *Sedum* sp*.* | 2 |
|  | *Sinningia speciose* | 1 |
|  | *Solanum tuberosum* | 3 |
|  | *Verbena x hybrida* | 4 |
|  | *Vinca minor* | 2 |
|  | *Viola* sp. | 3 |

^1^ Potential new hosts to the respective species are marked with an *.
