# Supplementary material for: Phytophthora Diversity in Pennsylvania Nurseries and Greenhouses Inferred from Clinical Samples Collected over Four Decades
Source: Microorganisms. 2020 Jul 16;8(7):1056. doi: 10.3390/microorganisms8071056 (PMC7409235; doi:10.3390/microorganisms8071056)
Supplement: Supplementary file 1 [file microorganisms-08-01056-s001.zip › Supplementary Table S3.doc]

Supplementary Table S3: Plants associated with Clade 2 species.

| Species | Host^1^ | # of isolates |
| --- | --- | --- |
| *P. tropicalis* (N=19) | *Antirrhinum majus* | 2 |
|  | *Epipremnum aureum* | 2 |
|  | *Fuchsia hybrida* | 1 |
|  | *Hedera helix* | 10 |
|  | *Lavandula* spp*.* * | 3 |
|  | *Pelargonium domesticum* * | 1 |
| *P. citrophthora* (N=42) | *Abies* spp*.* | 4 |
|  | *Buxus* sp*.* | 1 |
|  | *Calibrachoa* sp*.* * | 2 |
|  | *Cotoneaster* sp*.* | 1 |
|  | *Euonymus fortune* | 2 |
|  | *Hedera helix* | 1 |
|  | *Ilex* sp*.* | 1 |
|  | *Kalmia latifolia* | 2 |
|  | *Lamium* sp*.* * | 2 |
|  | *Lavandula officinalis* | 1 |
|  | *Myrica pensylvanica* | 1 |
|  | *Pelargonium domesticum* | 1 |
|  | *Picea pungens* | 1 |
|  | *Pieris* spp*.* | 6 |
|  | *Pseudotsugae menziesii* * | 1 |
|  | *Rhododendron* spp. | 8 |
|  | *Sciadopitys verticillate* | 1 |
|  | *Syringa* sp*.* | 3 |
|  | *Taxus* sp*.* | 2 |
|  | *Vinca minor* * | 1 |
| *P. capsici* (N= 32) | *Capsicum* spp*.* | 13 |
|  | *Citrullus* sp*.* | 1 |
|  | *Cucumis melo* | 1 |
|  | *Cucurbita* spp*.* | 8 |
|  | *Euonymus* sp. * | 1 |
|  | *Euphorbia pulcherrima* * | 6 |
|  | *Solanum lycopersicon* | 1 |
|  | *Malus sylvestris* | 1 |
| *P. capsici-like* (N=20) | *Capsicum annuum* | 2 |
|  | *Chrysanthemum* sp*.* | 1 |
|  | *Cucurbita maxima* | 4 |
|  | *Epipremnum aureum* | 7 |
|  | *Euphorbia pulcherrima* | 4 |
|  | *Solanum lycopersicon* | 1 |
|  | *Pseudotsuga menziesii* | 1 |
| *P. caryae* (N=8) | *Amelanchier* sp*.** | 1 |
|  | *Rhododendron* spp*.* | 7 |
| *P. multivora* (N=1) | *Vinca* sp*.* * | 1 |
| *P. plurivora* (N=80) | *Abies fraseri* | 8 |
|  | *Chamaecyparis lawsoniana* | 1 |
|  | *Kalmia latifolia* | 5 |
|  | *Picea* sp*.* | 1 |
|  | *Pieris japonica* | 3 |
|  | *Pinus strobus* | 2 |
|  | *Pseudotsuga menziesii* | 2 |
|  | *Rhododendron* spp*.* | 56 |
|  | *Unlisted* | 2 |
| *P. pini* (N=95) | *Abies* spp*.* | 8 |
|  | *Chamaecyparis lawsoniana* * | 3 |
|  | *Cornus florida* * | 3 |
|  | *Cucurbita pepo* * | 1 |
|  | *Ilex* spp*.* * | 3 |
|  | *Juglans nigra* * | 1 |
|  | *Kalmia latifolia* * | 1 |
|  | *Lonicera* sp*.* * | 1 |
|  | *Miscanthus* sp*.* * | 1 |
|  | *Picea* spp*.* * | 3 |
|  | *Pieris japonica* * | 4 |
|  | *Pinus strobus* | 3 |
|  | *Pseudotsuga menziesii* * | 3 |
|  | *Rhododendron* spp*.* | 57 |
|  | *Robinia pseudoacacia* * | 1 |
|  | *Sciadopitys verticillata* * | 2 |
| *P. bishii* (N=1) | *Rhododendron* sp*.* | 1 |

^1^ Potential new hosts are marked with an *.
