# Supplementary material for: Phytophthora Diversity in Pennsylvania Nurseries and Greenhouses Inferred from Clinical Samples Collected over Four Decades
Source: Microorganisms. 2020 Jul 16;8(7):1056. doi: 10.3390/microorganisms8071056 (PMC7409235; doi:10.3390/microorganisms8071056)
Supplement: Supplementary file 1 [file microorganisms-08-01056-s001.zip › Supplementary Table S4.doc]

Supplementary Table S4: Plants associated with Clades 4 and 5 species.

| Species | Host^1^ | # of isolates |
| --- | --- | --- |
| Clade 4 | | |
| *P. palmivora* (N=34) | *Calibrachoa sp.* * | 10 |
|  | *Chamaedorea elegans* | 3 |
|  | *Dracaena sp.* | 1 |
|  | *Fuchsia hybrid* * | 4 |
|  | *Hedera helix* | 14 |
|  | *Pescatoria sp* * | 1 |
|  | *Syringa sp.* | 1 |
| Clade 5 | | |
| *P. heveae* (N=2) | *Rhododendron* spp. | 2 |

^1^ Potential new hosts are marked with an *.
