# Supplementary material for: Phytophthora Diversity in Pennsylvania Nurseries and Greenhouses Inferred from Clinical Samples Collected over Four Decades
Source: Microorganisms. 2020 Jul 16;8(7):1056. doi: 10.3390/microorganisms8071056 (PMC7409235; doi:10.3390/microorganisms8071056)
Supplement: Supplementary file 1 [file microorganisms-08-01056-s001.zip › Supplementary Table S5.doc]

Supplementary Table S5: Plants associated with Clade 6 species.

| Species | Host^1^ | # of isolates |
| --- | --- | --- |
| *P. megasperma* (N=8) | *Castanea* sp*.* | 1 |
|  | *Forsythia* sp*.* * | 1 |
|  | *Picea pungens* | 1 |
|  | *Pinus strobus* | 1 |
|  | *Pseudotsuga menziesii* | 2 |
|  | *Rhododendron* sp*.* * | 1 |
|  | *Taxus* sp*.* * | 1 |
| *P. chlamydospora* (N=12) | *Abies fraseri* * | 1 |
|  | *Acer saccharum* * | 1 |
|  | *Ilex x meservae* * | 2 |
|  | *Rhododendron* sp*.* | 4 |
|  | *Sorbus americana* * | 1 |
|  | *Taxus* sp*.* | 1 |
|  | *Thuja* sp*.* * | 1 |
|  | *Tsuga* sp*.* * | 1 |
| *P. sp. personii* (N=1) | *Picea pungens** | 1 |
| *P. xstagnum* (N=1) | *Pseudotsuga menziesii* * | 1 |

^1^ Potential new hosts are marked with an (*).
