# Supplementary material for: Phytophthora Diversity in Pennsylvania Nurseries and Greenhouses Inferred from Clinical Samples Collected over Four Decades
Source: Microorganisms. 2020 Jul 16;8(7):1056. doi: 10.3390/microorganisms8071056 (PMC7409235; doi:10.3390/microorganisms8071056)
Supplement: Supplementary file 1 [file microorganisms-08-01056-s001.zip › Supplementary Table S6.doc]

Supplementary Table S6: Plants associated with Clade 7 species.

| Species | Host^1^ | # of isolates |
| --- | --- | --- |
| *P. cambivora* (N=10) | *Abies spp.* | 3 |
|  | *Pieris japonica* | 2 |
|  | *Pseudotsuga menziesii* | 1 |
|  | *Rhododendron* sp. | 3 |
|  | *Sorbus Americana* | 1 |
| *P. abietivora* (N=5) | *Abies fraseri* | 2 |
|  | *Tsuga canadensisI* * | 3 |
| *P. cinnamomi* (N=121) | *Abies* spp*.* | 17 |
|  | *Acer saccharum* | 1 |
|  | *Chamaecyparis* sp*.* | 1 |
|  | *Euonymus* sp*.* * | 1 |
|  | *Hydrangea quercifolia* * | 2 |
|  | *Ilex* spp*.* | 12 |
|  | *Juniperus* sp*.* | 1 |
|  | *Microbiota decussata* | 4 |
|  | *Oxydendrum arboretum* * | 1 |
|  | *Picea* spp*.* | 4 |
|  | *Pieris japonica* | 3 |
|  | *Pinus* spp*.* | 2 |
|  | *Prunus persica* | 1 |
|  | *Pseudotsuga menziesii* | 4 |
|  | *Rhododendron* spp. | 53 |
|  | *Sassafras* sp*.* * | 1 |
|  | *Sciadopitys verticillata* | 3 |
|  | *Syringa* sp*.* * | 1 |
|  | *Taxus* spp*.* | 6 |
|  | *Tsuga canadensis* | 2 |
|  | *Viburnum* sp*.* | 1 |
| *P. parvispora* (N=1) | *Lavandula* sp*.* * | 1 |
| *P. sojae* (N=8) | *Glycine max* | 8 |
| *P. niederhauserii* (N=3) | *Begonia x hiemalis* * | 2 |
|  | *Juniper horizontalis* * | 1 |

^1^ Potential new hosts are marked with an *.
