# Supplementary material for: Phytophthora Diversity in Pennsylvania Nurseries and Greenhouses Inferred from Clinical Samples Collected over Four Decades
Source: Microorganisms. 2020 Jul 16;8(7):1056. doi: 10.3390/microorganisms8071056 (PMC7409235; doi:10.3390/microorganisms8071056)
Supplement: Supplementary file 1 [file microorganisms-08-01056-s001.zip › Supplementary Table S7.doc]

Supplementary Table S7: Plants associated with Clade 8 species.

| Species | Host^1^ | # of isolates |
| --- | --- | --- |
| *P cryptogea* (N=8) | *Abies fraseri* | 1 |
|  | *Gerbera* sp*.* | 2 |
|  | *Solanum lycopersicon* | 4 |
|  | *Pieris japonica* | 1 |
| *P. drechsleri* (N=73) | *Ajuga* sp*.* * | 1 |
|  | *Antirrhinum majus* * | 1 |
|  | *Brachteanthus* sp*.* * | 1 |
|  | *Bracteantha bracteatum* * | 1 |
|  | *Calibrachoa* sp*.* * | 5 |
|  | *Chrysanthemum sp.* | 12 |
|  | *Citrullus* sp*.* | 1 |
|  | *Euphorbia pulcherrima* | 34 |
|  | *Gerbera* sp*.* | 3 |
|  | *Helichrysum bracheatum* | 2 |
|  | *Iberis sempervirens* * | 3 |
|  | *Lactuca sativa* | 1 |
|  | *Leucanthemum supurbum* * | 1 |
|  | *Osteospermum sp.* * | 1 |
|  | *Picea pungens* | 1 |
|  | *Pinus strobus* | 1 |
|  | *Pseudotsuga menziesii* | 1 |
|  | *Rhododendron spp.* | 1 |
|  | *Rudbeckia* sp*.* * | 1 |
|  | *Verbena x hybrida* * | 1 |
| *P. erythroseptica* (N=4) | *Solanum tuberosum* | 4 |
| *P. sansomeana (*N=19) | *Abies spp.* | 13 |
|  | *Picea mariana* * | 1 |
|  | *Prunus* sp*.* * | 1 |
|  | *Pseudotsuga menziesii* * | 3 |
|  | *Rubus idaeus* * | 1 |
| *P. pseudocryptogea* (N=4) | *Abies fraser* * | 1 |
|  | *Brassica oleracea* | 1 |
|  | *Pilea microphylla* * | 1 |
|  | *Pseudotsuga menziesii* * | 1 |
| *P.* sp*. kelmania* (N=288) | *Abies* spp. | 151 |
|  | *Cotoneaster* sp*.* * | 1 |
|  | *“Conifer”* | 1 |
|  | *Cucurbita pepo* * | 1 |
|  | *Echinops* sp*.* * | 1 |
|  | *Glycine max* * | 1 |
|  | *Heuchera x heucherella* * | 1 |
|  | *Ilex x meservae* * | 2 |
|  | *Picea* spp*.* | 21 |
|  | *Pinus* spp*.* * | 22 |
|  | *Pseudotsuga menziesii* * | 73 |
|  | *Quercus rubra* * | 1 |
|  | *Rhododendron* spp. * | 2 |
|  | *Rosmarinus officinalis* * | 1 |
|  | *Solanum tuberosum* * | 1 |
|  | *Spinacia oleracea* * | 1 |
|  | *Taxus* sp*.* * | 1 |
|  | *Thuja* sp*.* * | 2 |
|  | *Tsuga canadensis* * | 3 |
|  | *Viburnum setigerium* * | 1 |
| *P. foliorum* (N=2) | *Rhododendron* spp*.* | 2 |

^1^ Potential new hosts are marked with an *.
