# Supplementary material for: Phytophthora Diversity in Pennsylvania Nurseries and Greenhouses Inferred from Clinical Samples Collected over Four Decades
Source: Microorganisms. 2020 Jul 16;8(7):1056. doi: 10.3390/microorganisms8071056 (PMC7409235; doi:10.3390/microorganisms8071056)
Supplement: Supplementary file 1 [file microorganisms-08-01056-s001.zip › Supplementary Table S8.doc]

Supplementary Table S8: Plants associated with Clade 9 species.

| Species | Host^1^ | # of isolates |
| --- | --- | --- |
| *P. irrigata* (N=1) | *Rhododendron* spp*.* | 1 |
| *P. chrysanthemi* (N=6) | *Chrysanthemum* spp*.* | 6 |
| *P. hydropathica* (N=6) | *Epipremnum aureum* * | 1 |
|  | *Mangifera indica* * | 1 |
|  | *Pseudotsuga menziesii* * | 1 |
|  | *Psidium littorale* * | 1 |
|  | *Rhododendron* spp*.* | 2 |

^1^ Potential new hosts are marked with an *.
