# Supplementary material for: Phytophthora Diversity in Pennsylvania Nurseries and Greenhouses Inferred from Clinical Samples Collected over Four Decades
Source: Microorganisms. 2020 Jul 16;8(7):1056. doi: 10.3390/microorganisms8071056 (PMC7409235; doi:10.3390/microorganisms8071056)
Supplement: Supplementary file 1 [file microorganisms-08-01056-s001.zip › Supplementary Table S9.doc]

Supplementary Table S9: *Phytophthora* species found by county. Numbers in parenthesis indicate the number of isolates found for each species.

| County | Number of Isolates | Number of Species | Species found |
| --- | --- | --- | --- |
| Adams | 26 | 8 | *P. cactorum (14), P. capsici (1), P. citrophthora (3), P. capsici-like (1), P. niederhauserii (1), P. pini (2), P. sojae (1), P.* sp. *kelmania (3)* |
| Allegheny | 23 | 12 | *P. bishii (1), P. cactorum (1), P. caryae (1), P. chlamydospora (3), P. chrysanthemi (1), P. cinnamomi (4), P. citrophthora (2), P. nicotianae (1), P. palmivora (2), P. pini (4), P. plurivora (1), P.* sp. *kelmania (2)* |
| Armstrong | 14 | 4 | *P. cactorum (8), P. pini (2), P. plurivora (1), P.* sp. *kelmania (3)* |
| Beaver | 4 | 3 | *P. capsici (2), P. capsici-like (1), P. cinnamomi (1)* |
| Bedford | 3 | 3 | *P. drechsleri (1), P. plurivora (1), P.* sp. *kelmania (1)* |
| Berks | 25 | 10 | *P. cactorum (3), P. caryae (1), P. chrysanthemi (1), P. cinnamomi (3), P. citrophthora (1), P. nicotianae (6), P. pini (2), P. plurivora (2), P. sojae (2), P.* sp. *kelmania (4)* |
| Blair | 18 | 7 | *P. cactorum (1), P. caryae (2), P. cinnamomi (2), P. nicotianae (1), P. pini (3), P. plurivora (8), P.* sp. *kelmani (1)* |
| Bradford | 8 | 4 | *P. cactorum (1), P. cinnamomi (1), P. pini (1), P.* sp*. kelmania (5)* |
| Bucks | 60 | 12 | *P. cambivora (1), P. chlamydospora (4), P. cinnamomi (15), P. citrophthora (1), P. drechsleri (8), P. capsici-like (3), P. nicotianae (5), P. palmivora (4), P. pini (3), P. plurivora (4), P.* sp. *kelmania (8), P. tropicalis (4)* |
| Butler | 3 | 3 | *P. megasperma (1), P. nicotianae (1), P. pini (1)* |
| Cambria | 7 | 7 | *P. cactorum (1), P. cinnamomi (1), P. nicotianae (1), P. palmivora (1), P. pini (1), P. pseudotsugae (1), P.* sp. *kelmania (1)* |
| Carbon | 14 | 7 | *P. cactorum (2), P. cambivora (1), P. citrophthora (1), P. pini (2), P. plurivora (2), P. sansomeana (1), P.* sp. *kelmania (5)* |
| Centre | 11 | 5 | *P. cactorum (1), P. cinnamomi (2), P. pini (2), P. plurivora (1), P.* sp. *kelmania (5)* |
| Chester | 28 | 9 | *P. cactorum (3), P. cinnamomi (8), P. citrophthora (1), P. drechsleri (3), P. nicotianae (1), P. pini (3), P. plurivora (4), P. sojae (1), P.* sp. *kelmania (4)* |
| Clearfield | 4 | 4 | *P. abietivora (1), P. chlamydospora (1), P. pini (1), P. sansomeana (1)* |
| Clinton | 4 | 3 | *P. citrophthora (1), P. drechsleri (2), P. tropicalis (1)* |
| Columbia | 28 | 16 | *P. abietivora (1), P. cactorum (2), capsici (1), P. capsici-like (3), P. caryae (1), P. cinnamomi (3), P. drechsleri (1), P. hydropathica (1), P. megasperma (1), P. nicotianae (1), P. palmivora (2), P. pini (3), P. plurivora (1), P. pseudocryptogea (1), P.* sp. *kelmania (5), P. tropicalis (1)* |
| Crawford | 8 | 2 | *P. cactorum (1), P. sp. kelmania (7)* |
| Cumberland | 42 | 13 | *P. cactorum (4), P. cambivora (1), P. caryae (1), P. cinnamomi (6), P. citrophthora (2), P. cryptogea (3), P. drechsleri (5), P. nicotianae (8), P. palmivora (2), parvispora (1), P. pini (2), P. plurivora (3), P.* sp. *kelmania (4)* |
| Dauphin | 35 | 9 | *P. cactorum (4), P. cinnamomi (11), P. drechsleri (1), P. nicotianae (7), P. pini (3), P. plurivora (4), P. sansomeana (1), P.* sp. *kelmania (3), P.* sp*.[juniper] (1)* |
| Delaware | 6 | 3 | *P. cinnamomi (2), P. hydropathica (3), P. nicotianae (1),* |
| Erie | 4 | 3 | *P. caryae (1), P. cinnamomi (1), P.* sp. *kelmania (2)* |
| Fayette | 20 | 6 | *P. capsici (5), P. capsici-like (3), P. cinnamomi (2), P. drechsleri (8), P. plurivora (1), P.* sp. *kelmania (1)* |
| Franklin | 16 | 9 | *P. capsici (2), P. cinnamomi (2), P. citricola* complex *(1) P. citrophthora (1), P. drechsleri (1), P. nicotianae (5), P. palmivora (1), P. pini (2), P.* sp. *kelmania (1)* |
| Fulton | 1 | 1 | *P. cinnamomi (1)* |
| Greene | 1 | 1 | *P. citrophthora (1)* |
| Huntingdon | 2 | 1 | *P. sp. kelmania (2)* |
| Indiana | 76 | 13 | *P. abietivora (1), P. cactorum (11), P. chrysanthemi (2), P. cinnamomi (6), P. citrophthora (6), P. drechsleri (3), heveae (2), P. nicotianae (5), P. palmivora (1), P. pini (7), P. plurivora (5), P.* sp. *kelmania (25), P. tropicalis (2)* |
| Juniata | 8 | 4 | *P. drechsleri (1), P. pini (1), P. plurivora (1), P.* sp. *kelmania (5)* |
| Lackawanna | 23 | 7 | *P. cactorum (1), P. capsici (2), P. drechsleri (1), P. pini (3), P. plurivora (1), P.* sp. *kelmania (14), P. xstagnum (1)* |
| Lancaster | 95 | 20 | *P. cactorum (4), P. capsici (11), P. capsici-like (4), P. chrysanthemi (1), P. cinnamomi (6), P. citrophthora (4), P. cryptogea (1), P. drechsleri (7), P. foliorum (1), P. hedraiandra (2), P. hydropathica (1), P. nicotainae (15), P. palmivora (7), P. pini (5), P. plurivora (5), P. pseudocryptogea (1), P. sansomeana (1), P. sojae (2), P.* sp. *kelmania (11), P. tropicalis (5)* |
| Lawrence | 6 | 6 | *P. cinnamomi (1), P. megasperma (1), P. nicotianae (1), P. plurivora (1), P.* sp*. kelmania (1), P.* sp*. personii (1)* |
| Lebanon | 17 | 8 | *P. drechsleri (1), P. capsici-like (1), P. nicotianae (3), P. pini (2), P. sansomeana (1), P. sojae (1), P.* sp. *kelmania (6), P. tropicalis (2)* |
| Lehigh | 24 | 10 | *P. abietivora (1), P. cactorum (3), P. chrysanthemi (1), P. cinnamomi (2), P. drechsleri (4), P. erythroseptica (1), P. nicotianae (6), P. pini (3), P.* sp. *kelmania (2), P. tropicalis (1)* |
| Luzerne | 121 | 15 | *P. cactorum (8), P. cambivora (1), P. capsici (3), P. chlamydospora (1), P. cinnamomi (2), P. citrophthora (1), P. drechsleri (1), foliorum (1), megasperma (1), P. nicotianae (2), P. palmivora (1), P. pini (12), P. plurivora (9), P. sansomeana (7), P.* sp. *kelmania (71)* |
| Lycoming | 37 | 13 | *P. abietivora (1), P. cactorum (1), P. cambivora (1), P. cinnamomi (3), P. citrophthora (4), P. cryptogea (2), P. drechsleri (1), P. megasperma (1), P. capsici-like (1), P. palmivora (3), P. pini (3), P. plurivora (1), P.* sp. *kelmania (15)* |
| Mercer | 1 | 1 | *P. citrophthora (1)* |
| Mifflin | 1 | 1 | *P.* sp*. kelmania (1)* |
| Monroe | 16 | 9 | *P. cactorum (1), P. cambivora (1), P. cinnamomi (1), P. cryptogea (1), P. megasperma (1), P. nicotianae (2), P. pini (3), P. plurivora (5), P.* sp. *kelmania (1)* |
| Montgomery | 35 | 12 | *P. cambivora (1), P. capsici (1), P. chlamydospora (2), P. cinnamomi (7), P. citrophthora (3), P. irrigata (1), P. nicotianae (5), P. niederhaauserii (1), P. palmivora (3), P. plurivora (3), P. sansomeana (1), P.* sp. *kelmania (7)* |
| Montour | 8 | 5 | *P. cinnamomi (1), P. drechsleri (3), P. erythroseptica (1), P. nicotianae (1), P. pini (2)* |
| Northampton | 6 | 4 | *P. cactorum (1), drechsleri (2), P. nicotianae (2), P. pini (1)* |
| Northumberland | 7 | 6 | *P. cinnamomi (1), P. drechsleri (2), P. nicotianae (1), P. niederhauserii (1), P. sojae (1), P. tropicalis (1)* |
| Perry | 10 | 7 | *P. chlamydospora (1), P. cinnamomi (1), P. citrophthora (2), P. drechsleri (2), P. nicotianae (2), P. pini (1), P.* sp. *kelmania (1)* |
| Pike | 1 | 1 | *P. cambivora (1)* |
| Potter | 2 | 2 | *P. erythroseptica (1), P.* sp*. kelmania (1)* |
| Schuylkill | 38 | 10 | *P. cactorum (1), P. cinnamomi (5), P. drechsleri (5), P. capsici-like (2), P. nicotianae (7), P. palmivora (3), P. pini (2), P. plurivora (2), P.* sp. *kelmania (9), P. tropicalis (2)* |
| Snyder | 14 | 8 | *P. cactorum (2), P. capsici (1), P. cinnamomi (2), P. citrophthora (1), P. drechsleri (1), P. nicotianae (1), P. pini (1), P.* sp. *kelmania (5)* |
| Somerset | 7 | 6 | *P. cactorum (1), P. cryptogea (1), P. drechsleri (1), P. nicotianae (1), P. pini (1), P. plurivora (2)* |
| Sullivan | 7 | 4 | *P. citrophthora (1), P. pini (1), P. plurivora (3), P.* sp. *kelmania (2)* |
| Susquehanna | 10 | 5 | *P. cactorum (1), P. megasperma (1), P. pini (1), P. sansomeana (1), P.* sp. *kelmania (6)* |
| Tioga | 2 | 2 | *P. nicotianae (1), P.* sp. *kelmania (1)* |
| Union | 29 | 7 | *P. cactorum (4), P. capsici (1), P. cinnamomi (6), P. drechsleri (1), P. erythroseptica (1), P. nicotianaae (1), P.* sp. *kelmania (15)* |
| Venango | 1 | 1 | *P. nicotianae (1)* |
| Washington | 5 | 4 | *P. cinnamomi (2), P. citrophthora (1), P. multivora (1), P.* sp. *kelmania (1)* |
| Wayne | 8 | 5 | *P. cactorum (3), P. cambivora (1), P. caryae (1), P. pini (1), P. plurivora (2)* |
| Westmoreland | 21 | 9 | *P. cactorum (1), P. cinnamomi (2), P. citrophthora (2), P. drechsleri (3), P. nicotianae (3), P. pini (2), P. plurivora (2), P. pseudocryptogea (1), P.* sp. *kelmania (5)* |
| Wyoming | 11 | 9 | *P. cactorum (1), P. cinnamomi (1), megasperma (1), P. nicotianae (1), P. palmivora (1), P. pini (1), P. pseudocryptogea (1), P. sansomeana (1), P.* sp. *kelmania (3)* |
| York | 39 | 12 | *P. cactorum (3), P. capsici (1), P. capsici-like (1), P. cinnamomi (3), P. citrophthora (1), P. drechsleri (3), P. nicotianae (7), P. palmivora (1), P. pini (4), P. plurivora (2), P. sansomeana (2), P.* sp. *kelmania (11)* |
| NA^1^ | 36 | 13 | *P. cactorum (12), P. cambivora (1), P. capsici (1), P. cinnamomi (4), P. citrophthora (1), P. drechsleri (1), P. hydropathica (1), P. nicotianae (5), P. palmivora (2), P. pini (1), P. plurivora (3), P. sansomeana (2), P.* sp. *kelmania (2)* |

^1^The origins of the samples in this group are not available.
