# Supplementary figures and images for: Phytophthora Diversity in Pennsylvania Nurseries and Greenhouses Inferred from Clinical Samples Collected over Four Decades
Source: Microorganisms. 2020 Jul 16;8(7):1056. doi: 10.3390/microorganisms8071056 (PMC7409235; doi:10.3390/microorganisms8071056)

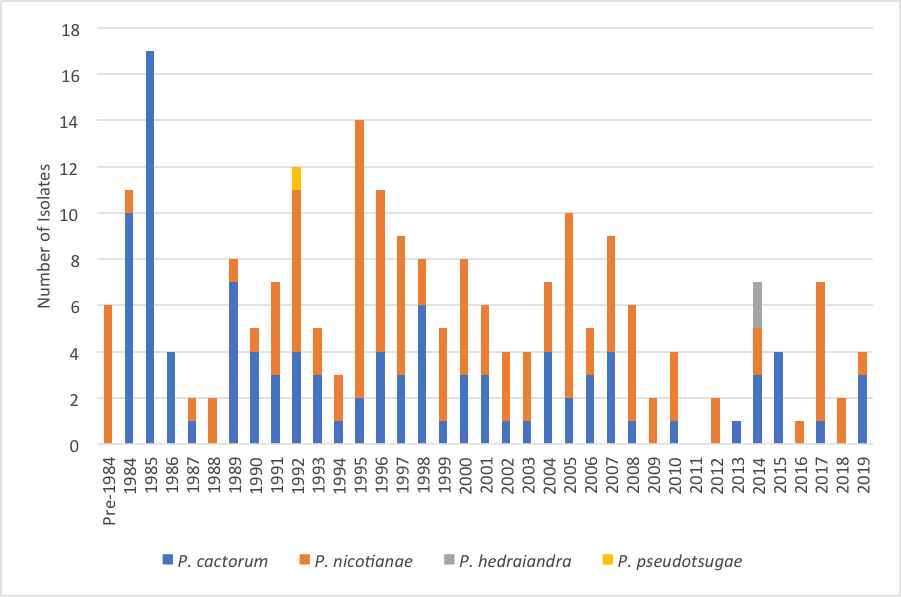

Supplement: Supplementary file 1 [file microorganisms-08-01056-s001.zip › FigureS1.png]

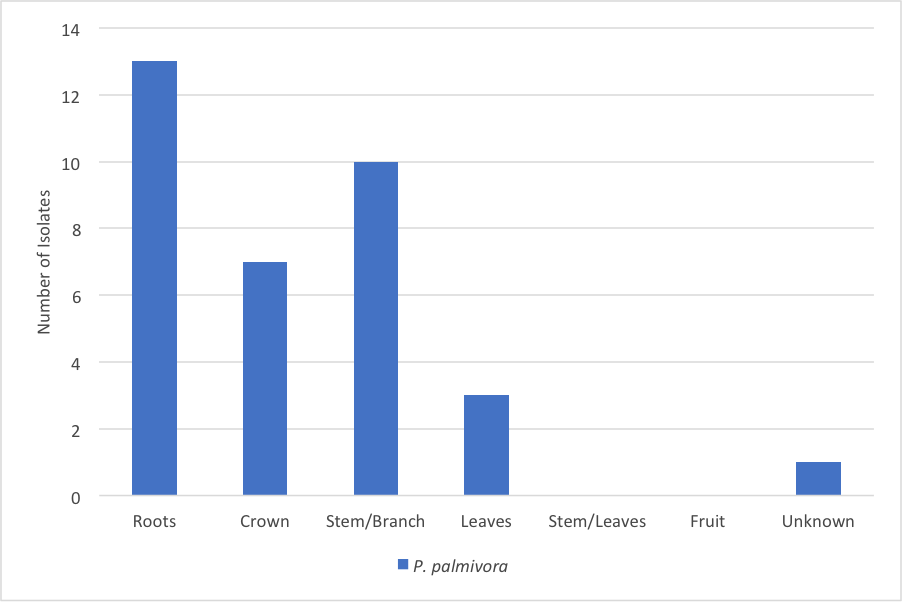

Supplement: Supplementary file 1 [file microorganisms-08-01056-s001.zip › FigureS10.png]

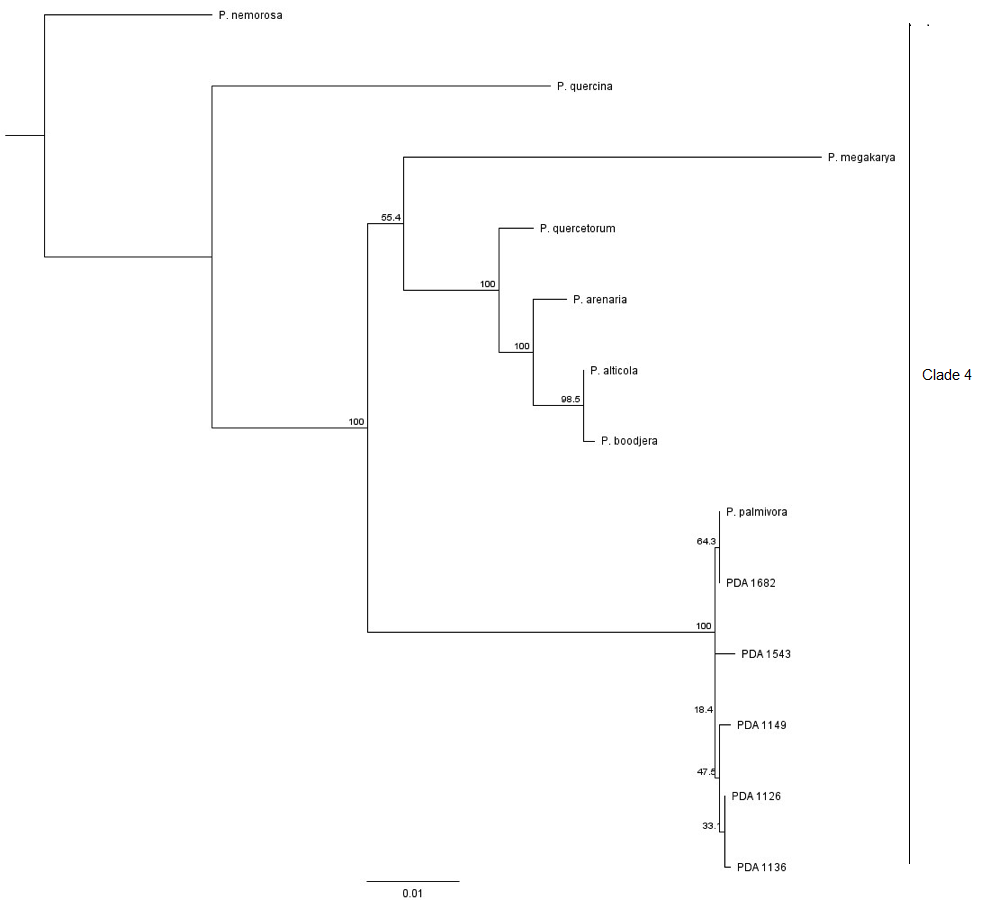

Supplement: Supplementary file 1 [file microorganisms-08-01056-s001.zip › FigureS11.png]

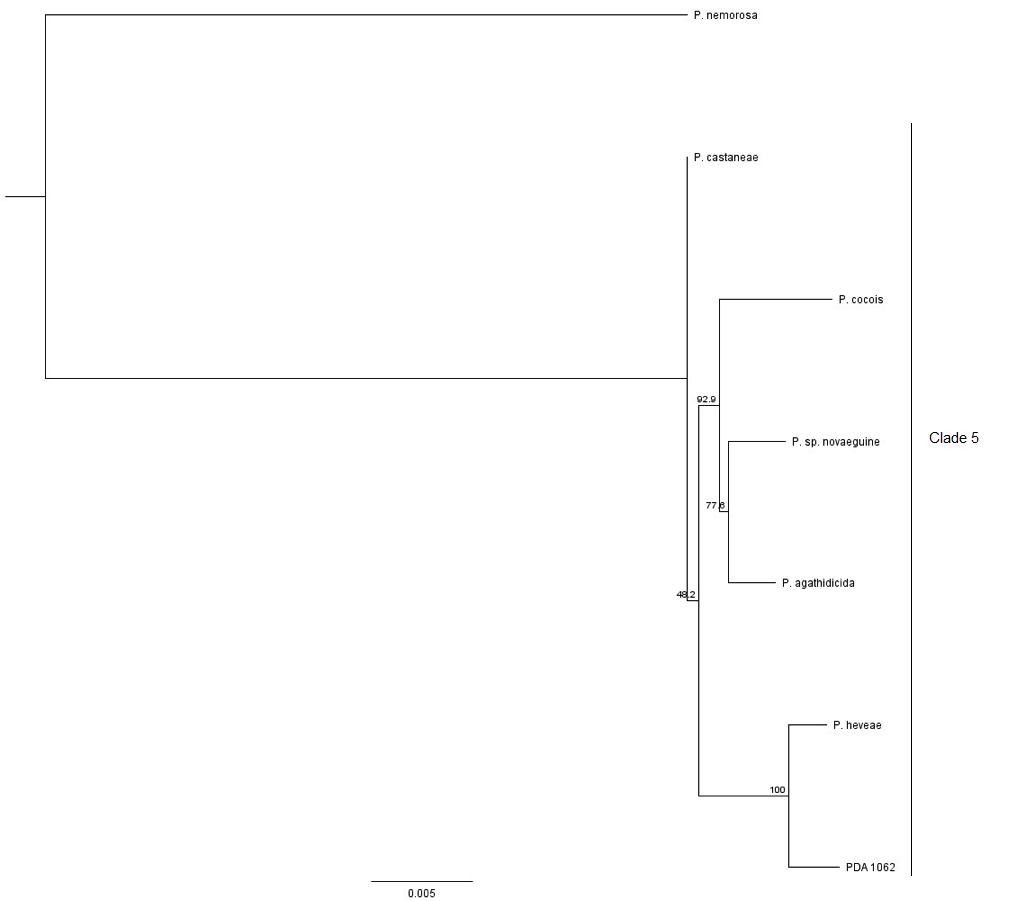

Supplement: Supplementary file 1 [file microorganisms-08-01056-s001.zip › FigureS12.png]

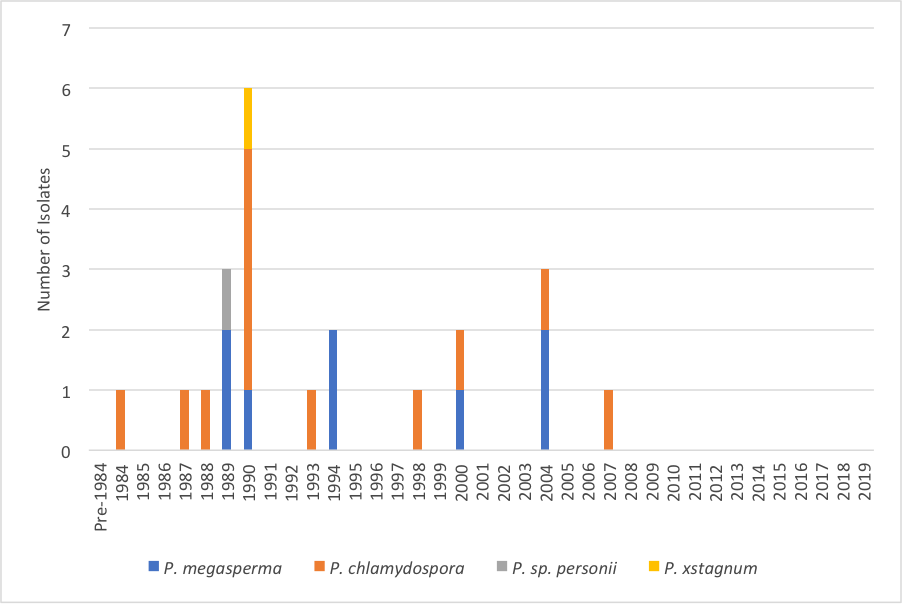

Supplement: Supplementary file 1 [file microorganisms-08-01056-s001.zip › FigureS13.png]

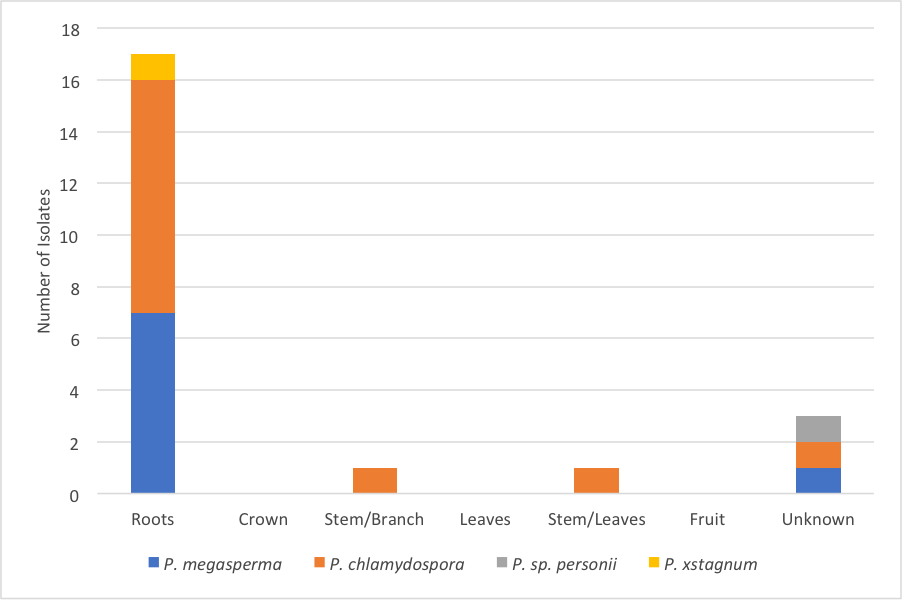

Supplement: Supplementary file 1 [file microorganisms-08-01056-s001.zip › FigureS14.png]

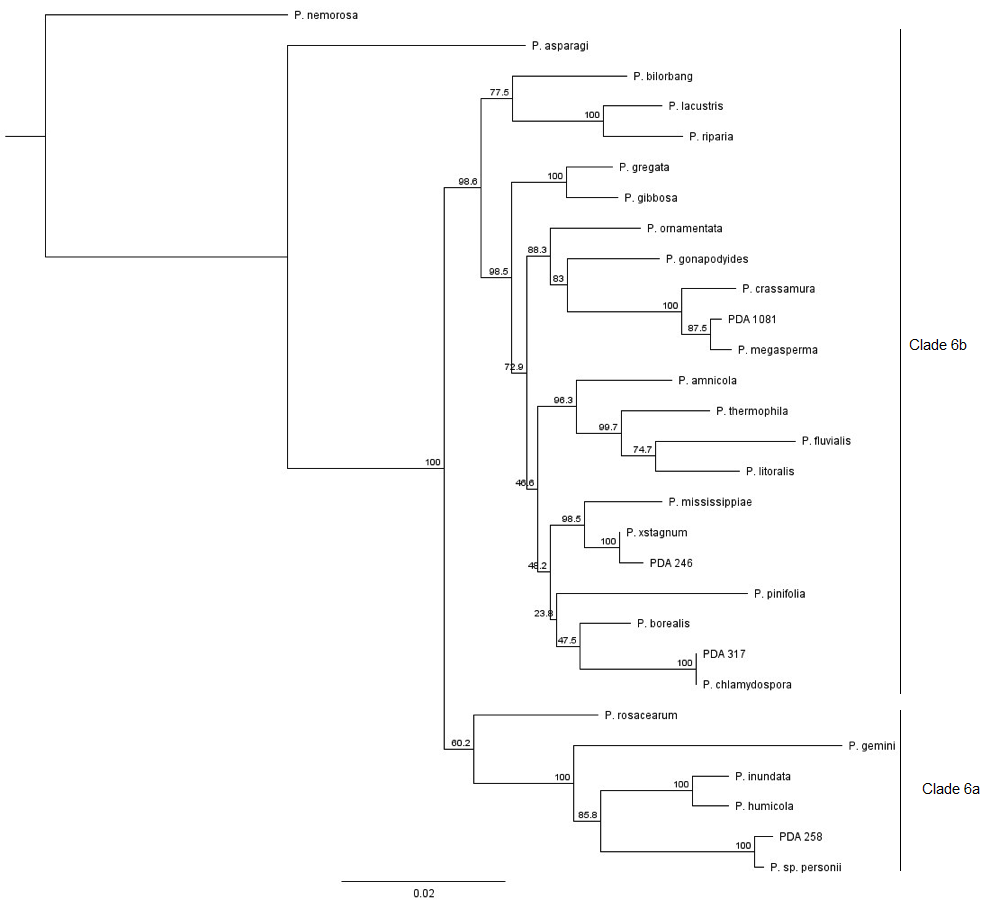

Supplement: Supplementary file 1 [file microorganisms-08-01056-s001.zip › FigureS15.png]

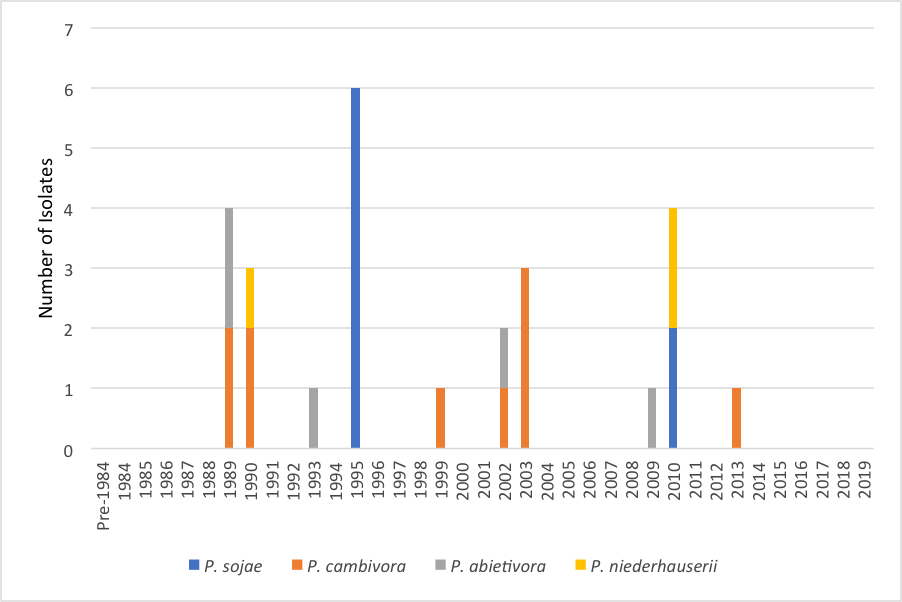

Supplement: Supplementary file 1 [file microorganisms-08-01056-s001.zip › FigureS16.png]

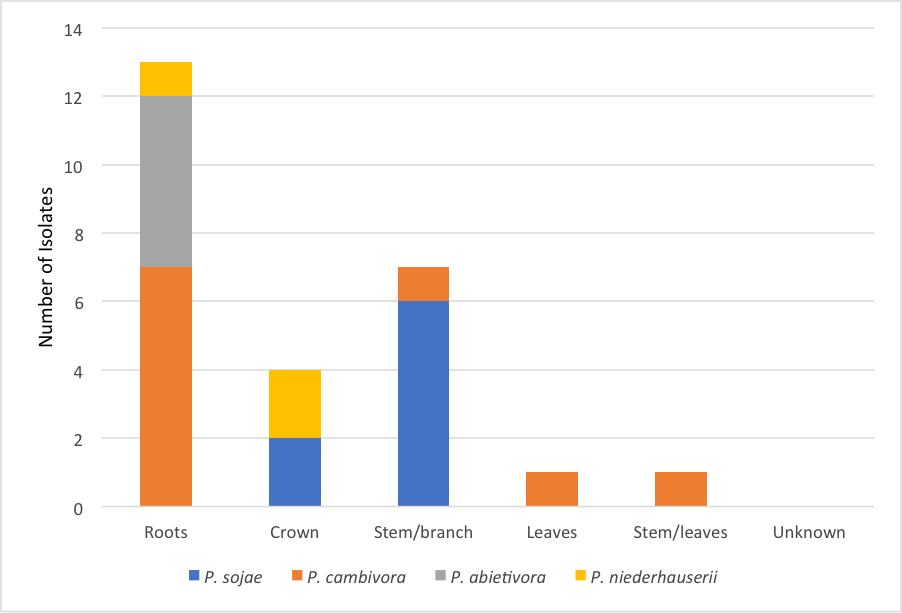

Supplement: Supplementary file 1 [file microorganisms-08-01056-s001.zip › FigureS17.png]

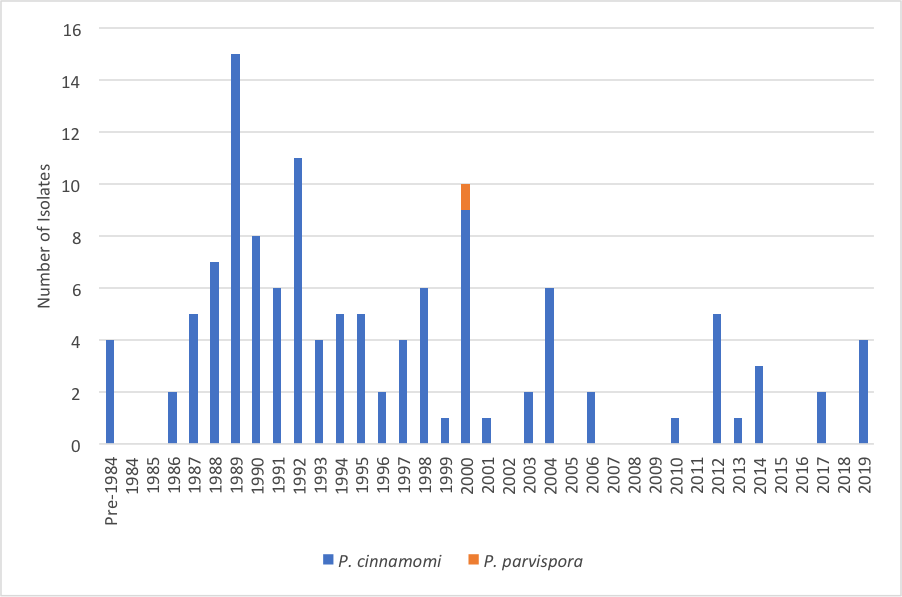

Supplement: Supplementary file 1 [file microorganisms-08-01056-s001.zip › FigureS18.png]

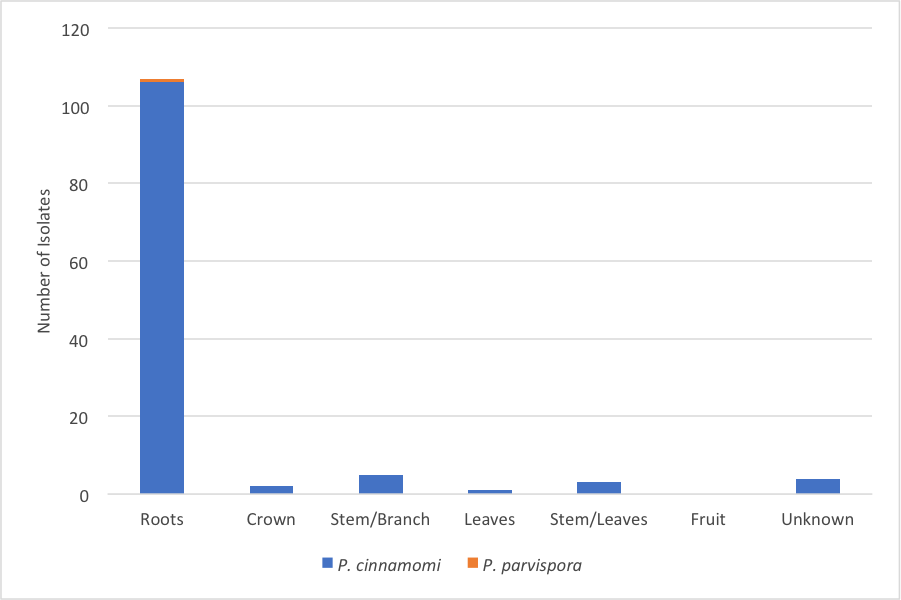

Supplement: Supplementary file 1 [file microorganisms-08-01056-s001.zip › FigureS19.png]

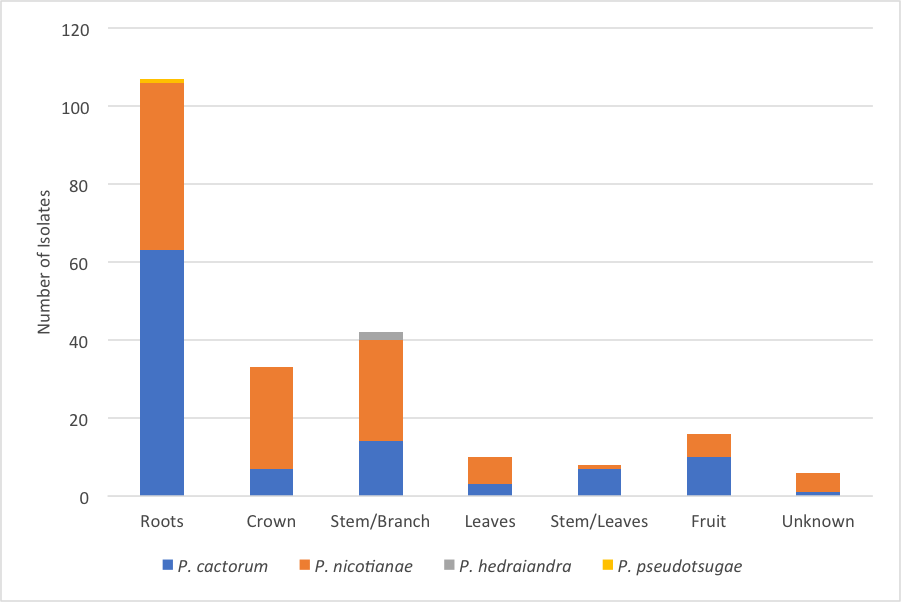

Supplement: Supplementary file 1 [file microorganisms-08-01056-s001.zip › FigureS2.png]

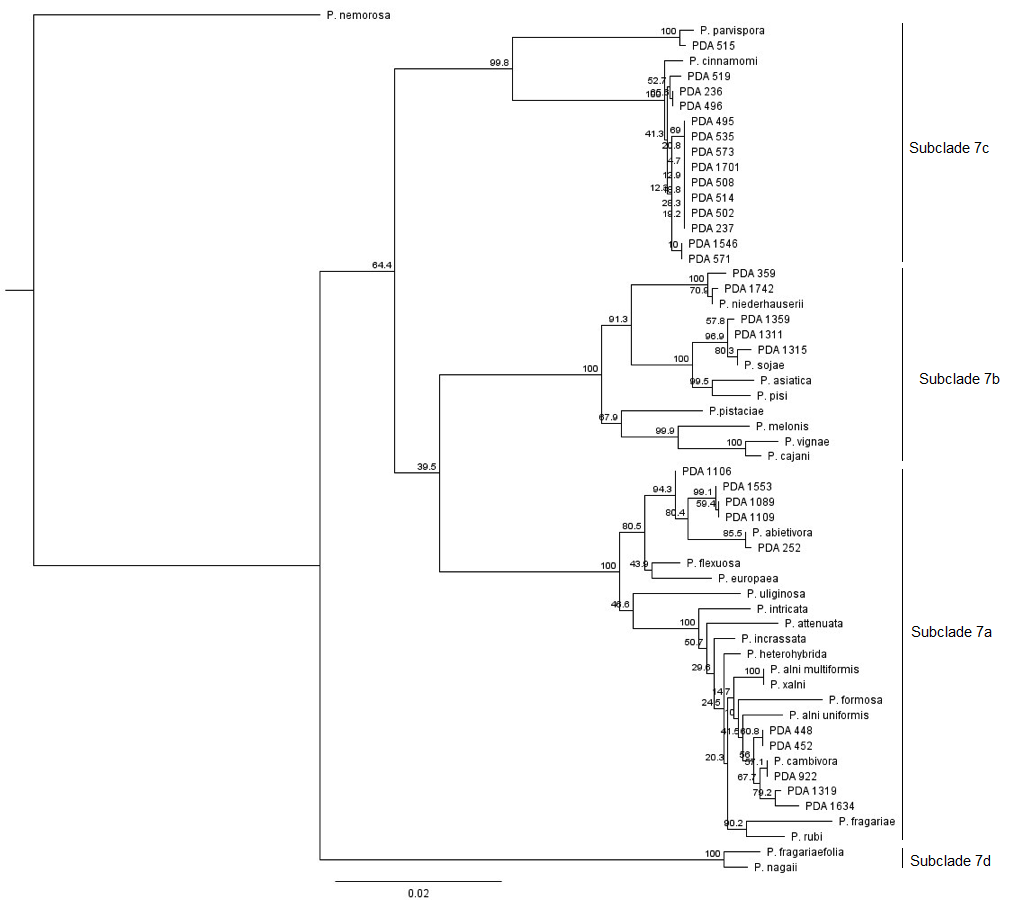

Supplement: Supplementary file 1 [file microorganisms-08-01056-s001.zip › FigureS20.png]

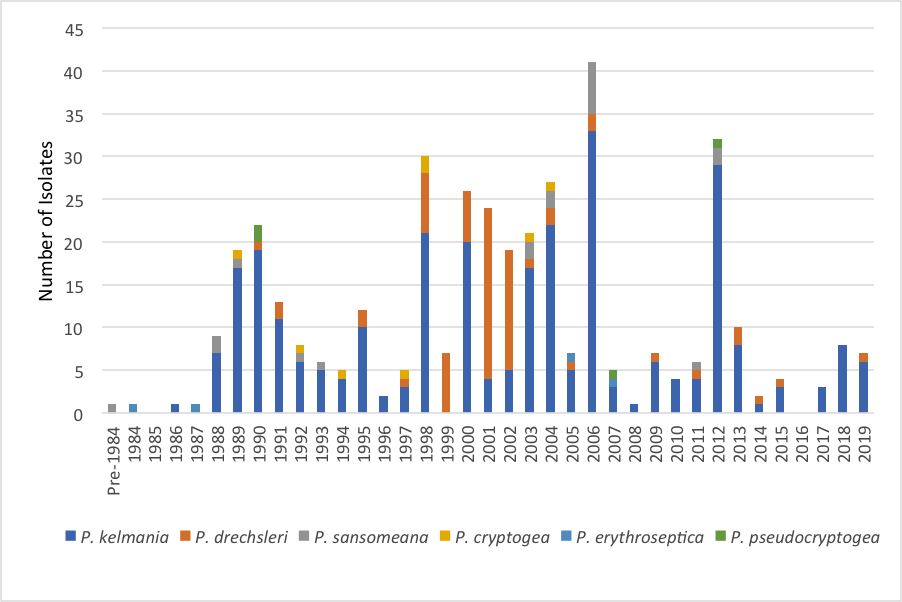

Supplement: Supplementary file 1 [file microorganisms-08-01056-s001.zip › FigureS21.png]

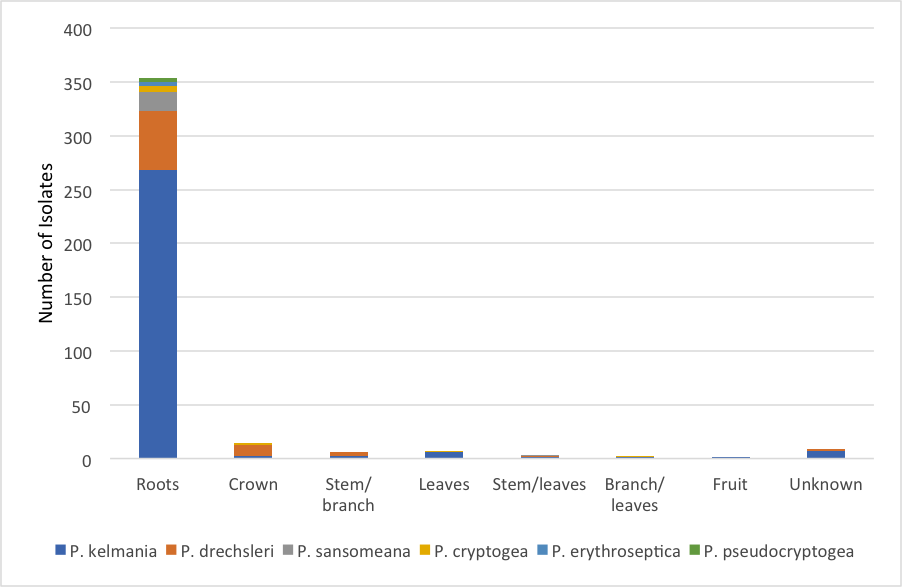

Supplement: Supplementary file 1 [file microorganisms-08-01056-s001.zip › FigureS22.png]

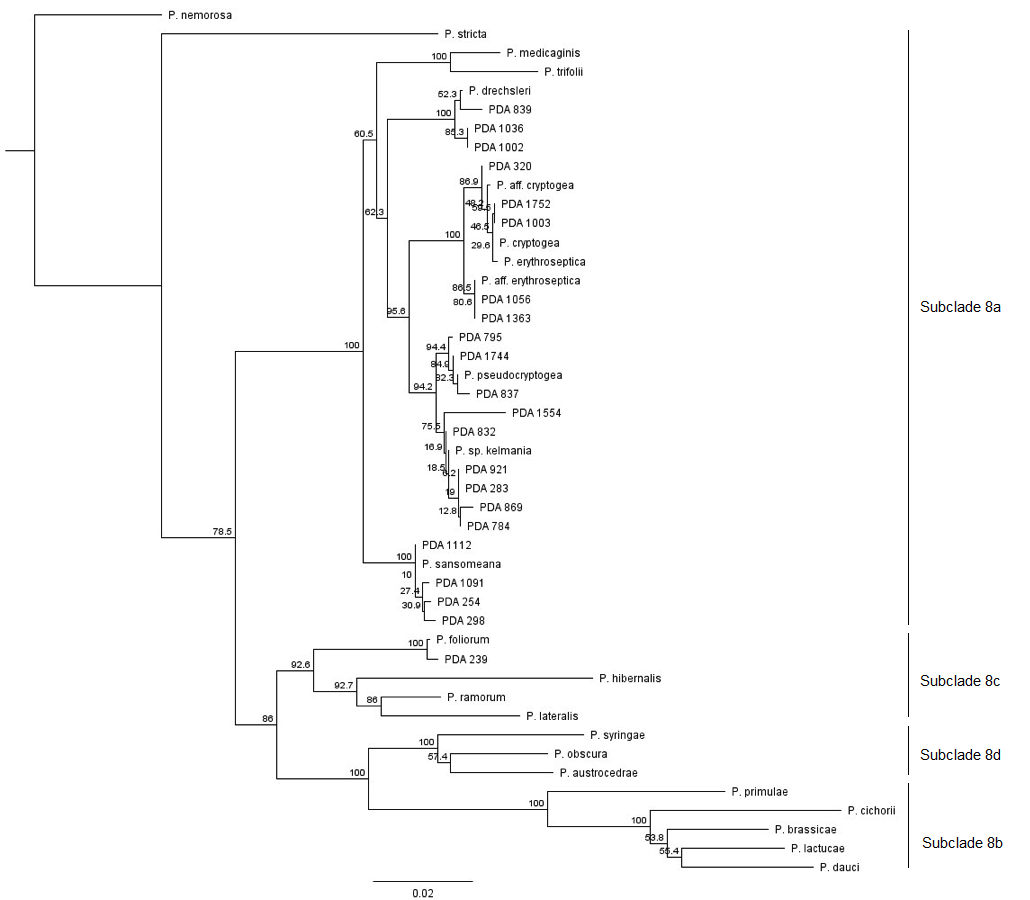

Supplement: Supplementary file 1 [file microorganisms-08-01056-s001.zip › FigureS23.png]

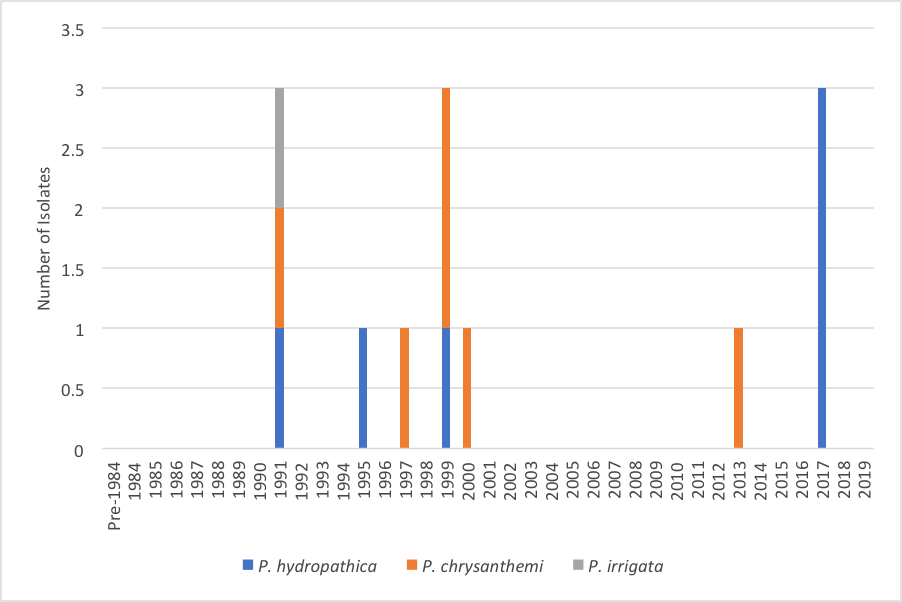

Supplement: Supplementary file 1 [file microorganisms-08-01056-s001.zip › FigureS24.png]

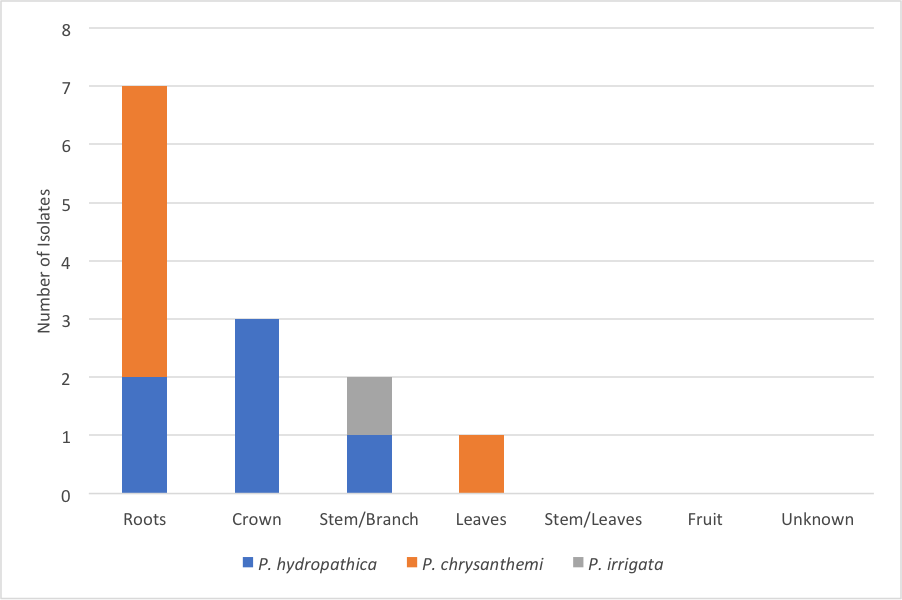

Supplement: Supplementary file 1 [file microorganisms-08-01056-s001.zip › FigureS25.png]

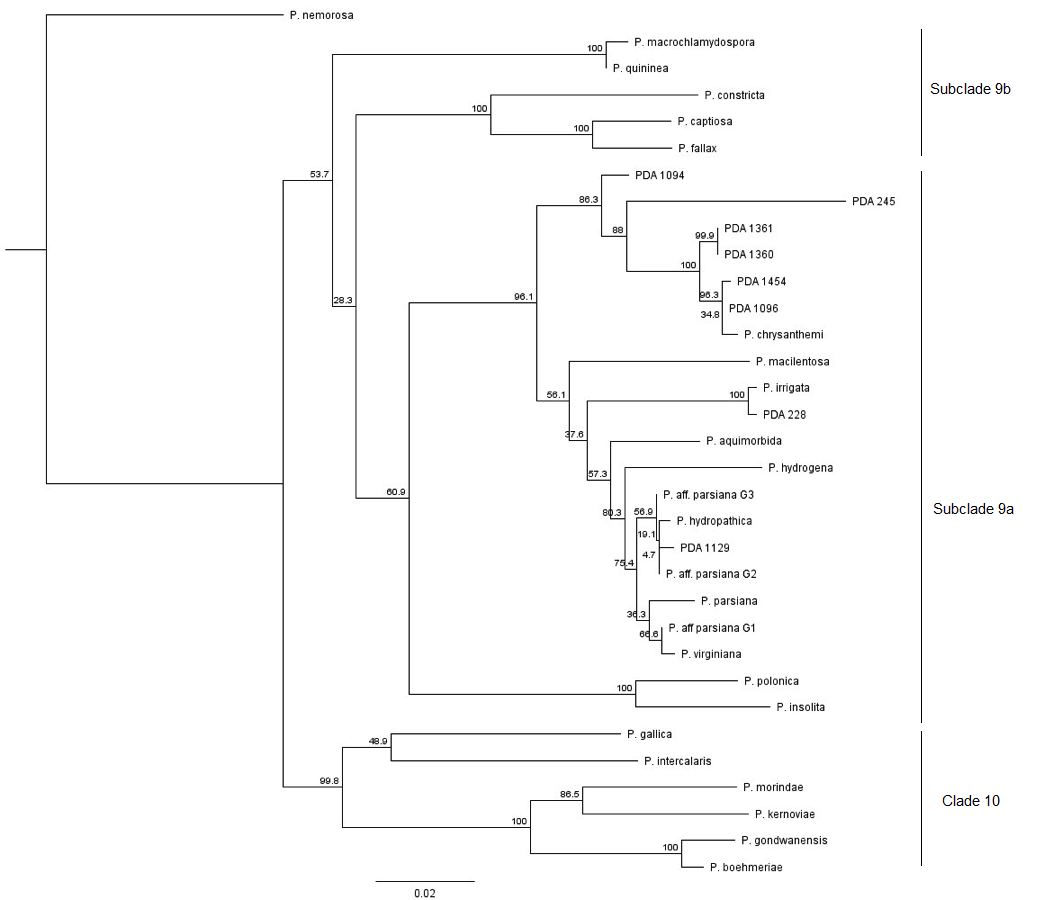

Supplement: Supplementary file 1 [file microorganisms-08-01056-s001.zip › FigureS26 (1).png]

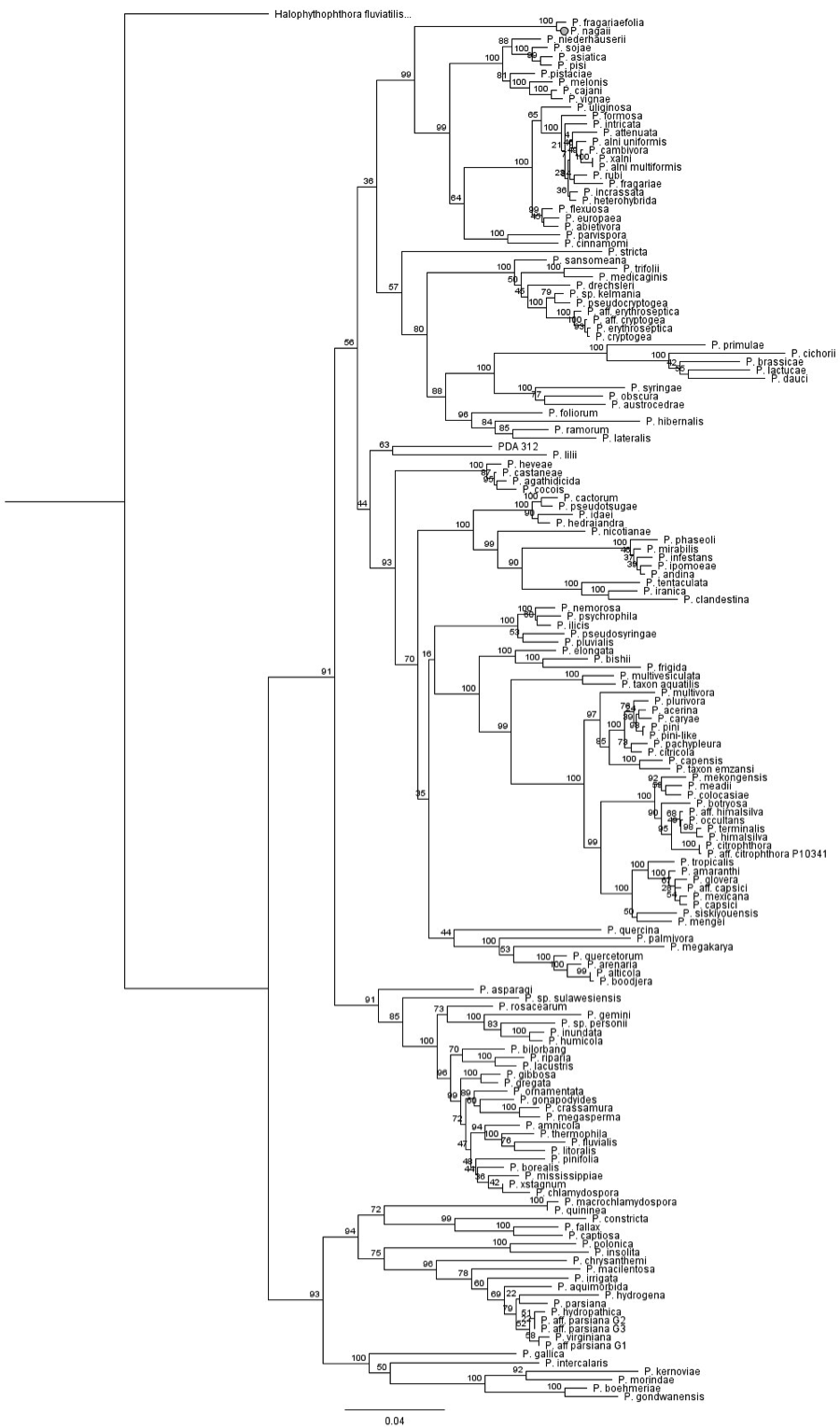

Supplement: Supplementary file 1 [file microorganisms-08-01056-s001.zip › FigureS27.png]

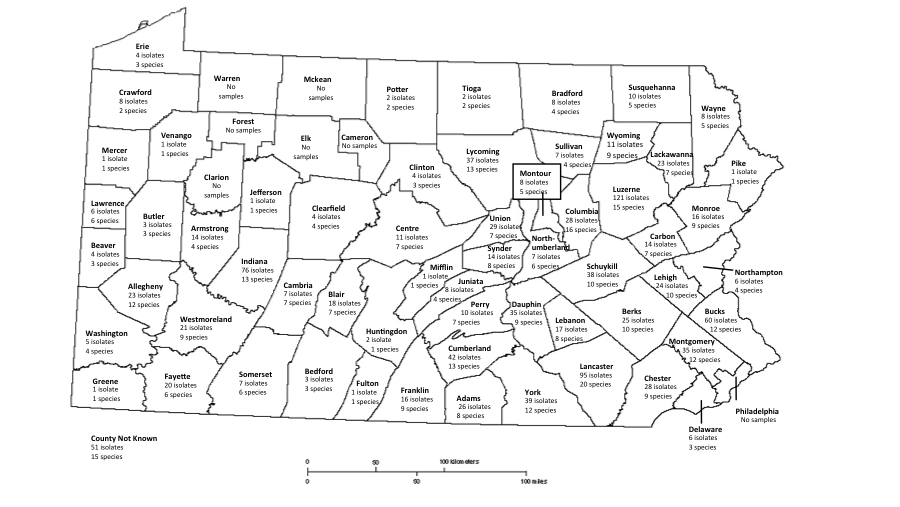

Supplement: Supplementary file 1 [file microorganisms-08-01056-s001.zip › FigureS28.png]

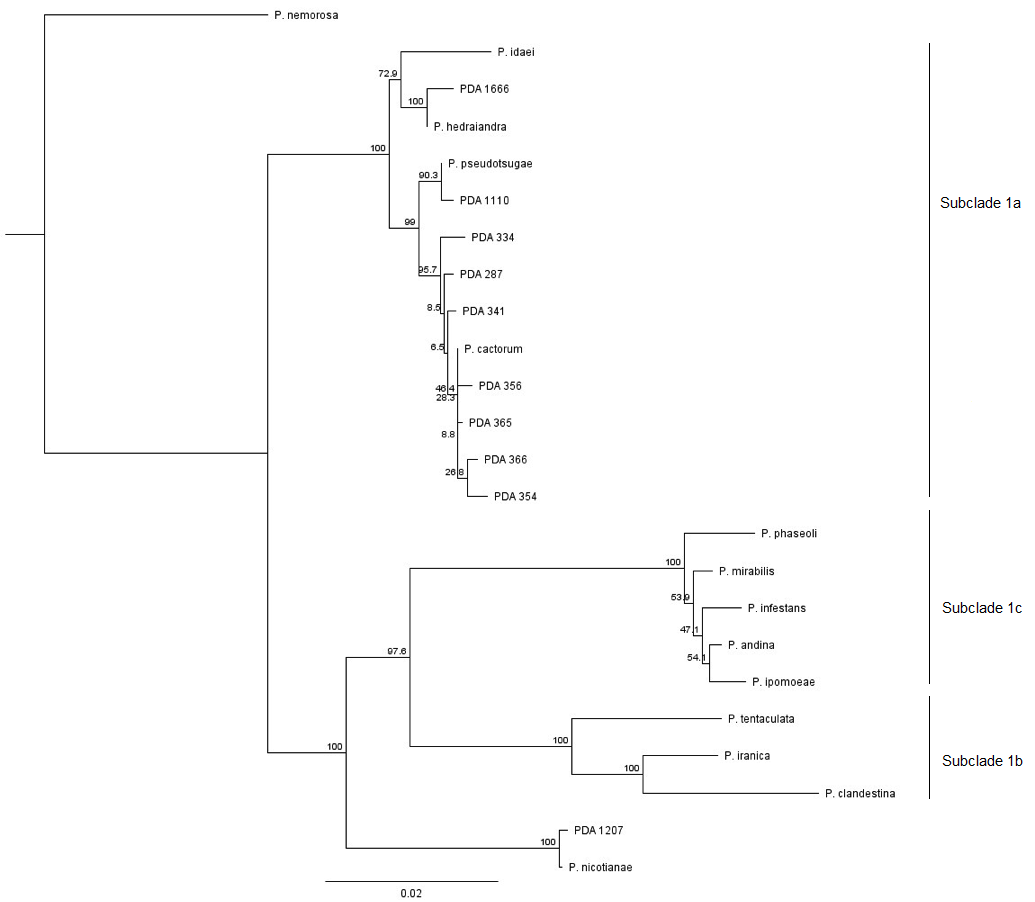

Supplement: Supplementary file 1 [file microorganisms-08-01056-s001.zip › FigureS3.png]

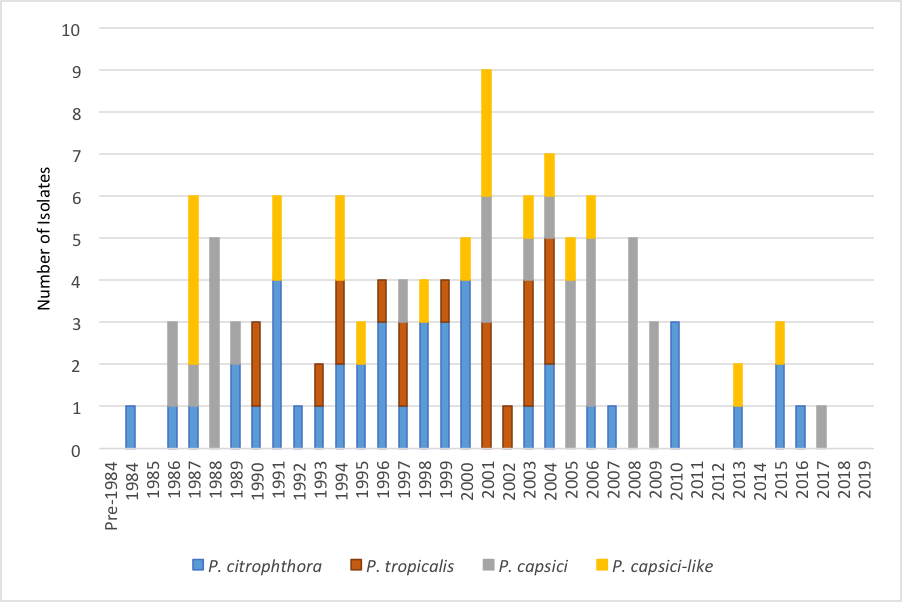

Supplement: Supplementary file 1 [file microorganisms-08-01056-s001.zip › FigureS4.png]

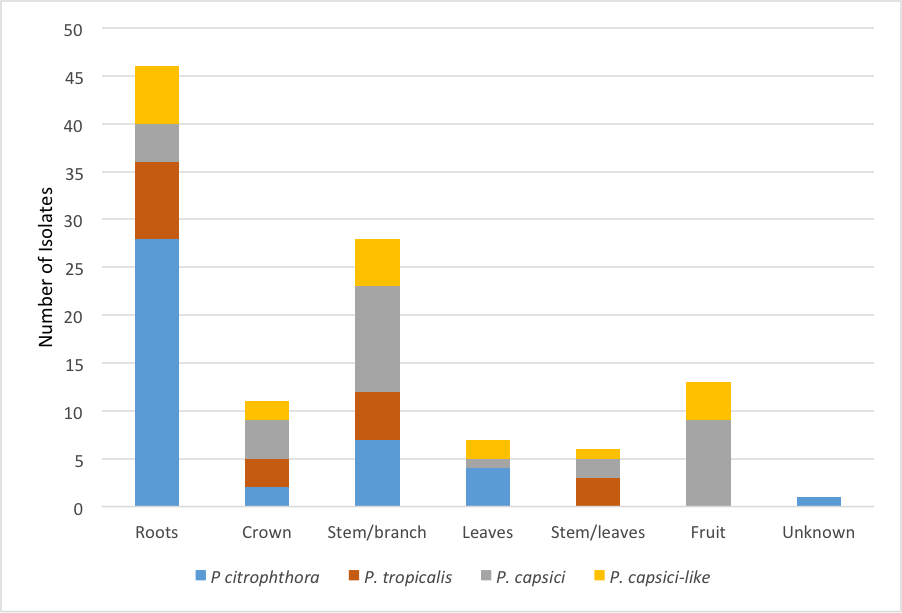

Supplement: Supplementary file 1 [file microorganisms-08-01056-s001.zip › FigureS5.png]

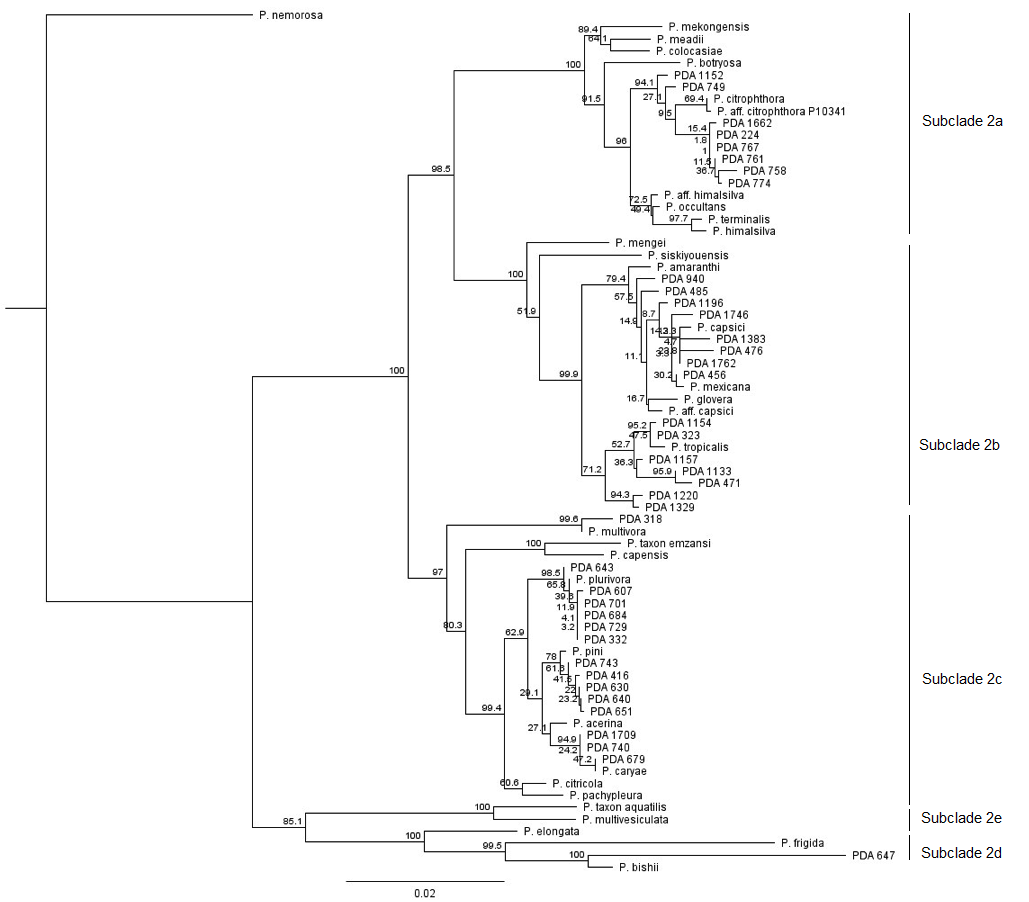

Supplement: Supplementary file 1 [file microorganisms-08-01056-s001.zip › FigureS6.png]

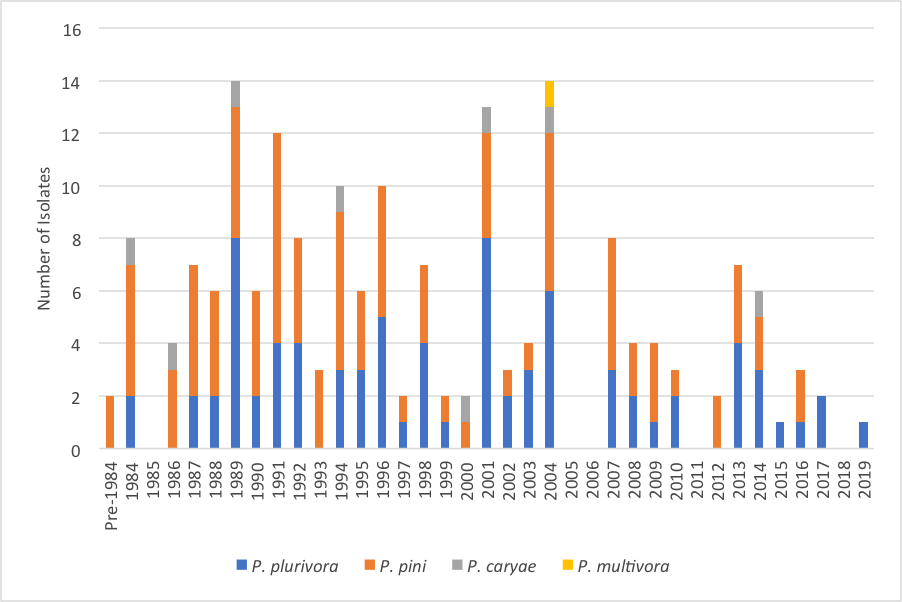

Supplement: Supplementary file 1 [file microorganisms-08-01056-s001.zip › FigureS7.png]

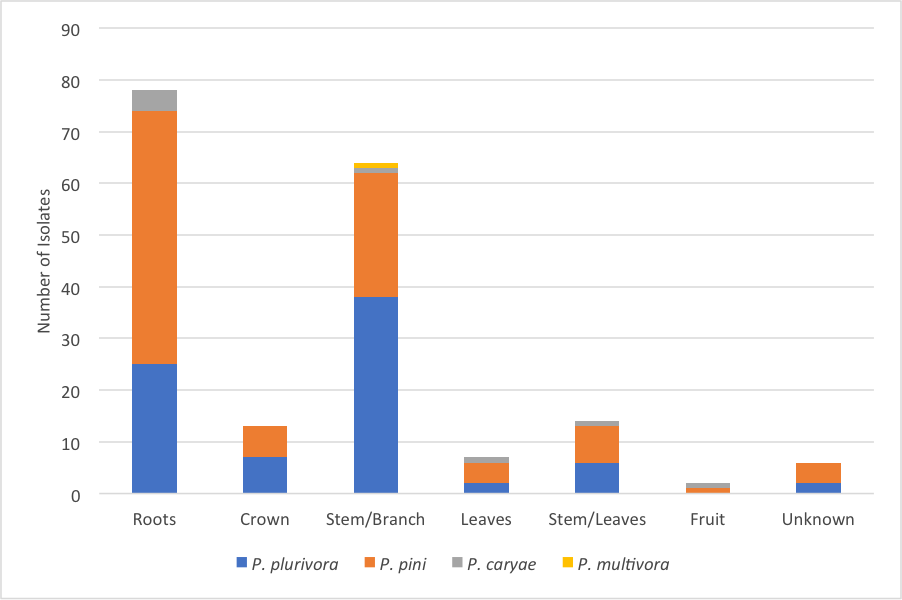

Supplement: Supplementary file 1 [file microorganisms-08-01056-s001.zip › FigureS8.png]

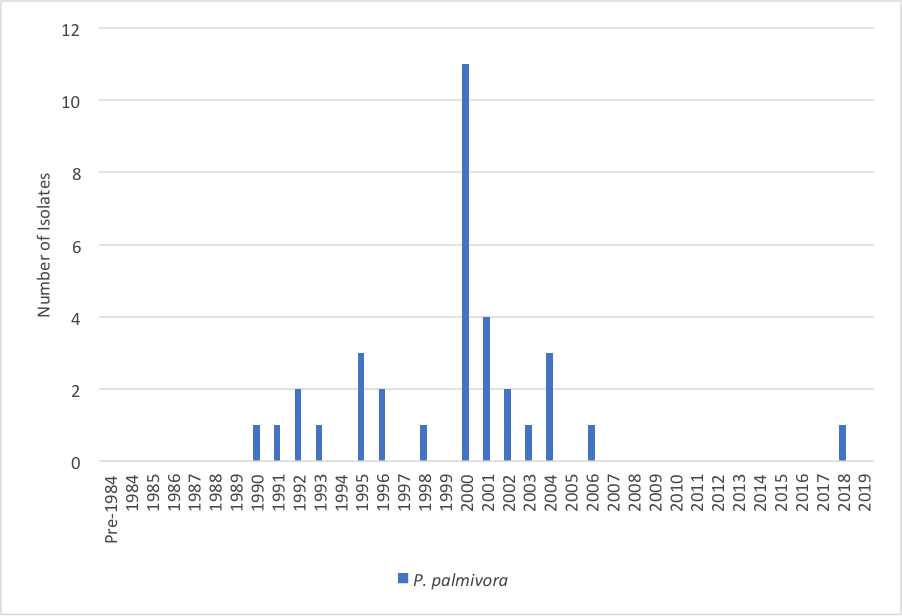

Supplement: Supplementary file 1 [file microorganisms-08-01056-s001.zip › FigureS9.png]
